# Mental disorders among workers in the healthcare industry: 2014 national health insurance data

**DOI:** 10.1186/s40557-018-0244-x

**Published:** 2018-05-03

**Authors:** Min-Seok Kim, Taeshik Kim, Dongwook Lee, Ji-hoo Yook, Yun-Chul Hong, Seung-Yup Lee, Jin-Ha Yoon, Mo-Yeol Kang

**Affiliations:** 1 Department of Preventive Medicine, Seoul National University College of Medicine, 103 Daehak-ro, Jongno-gu, Seoul 03080 Republic of Korea; 2 Department of Psychiatry, Uijeongbu St. Mary’s Hospital, College of Medicine, The Catholic University of Korea, 271 Cheonbo-ro, Uijeongbu, Gyeonggi-do Republic of Korea; 3 Department of Preventive Medicine, Yonsei University College of Medicine, 50-1 Yonsei-ro, Seodaemun-gu, Seoul 03722 Republic of Korea; 4 Department of Occupational and Environmental Medicine, College of Medicine, The Catholic University of Medicine Korea, 222, Banpo-daero, Seocho-gu, Seoul 06591 Republic of Korea

**Keywords:** Mood disorder, Anxiety disorder, Sleep disorder, Psychiatric disorders, Healthcare industry

## Abstract

**Background:**

Numerous studies have shown that healthcare professionals are exposed to psychological distress. However, since most of these studies assessed psychological distress using self-reporting questionnaires, the magnitude of the problem is largely unknown. We evaluated the risks of mood disorders, anxiety disorders, sleep disorders, and any psychiatric disorders in workers in healthcare industry using Korea National Health Insurance (NHI) claims data from 2014, which are based on actual diagnoses instead of self-evaluation.

**Methods:**

We used Korea 2014 NHI claims data and classified employees as workers in the healthcare industry, based on companies in the NHI database that were registered with hospitals, clinics, public healthcare, and other medical services. To estimate the standardized prevalence of the selected mental health disorders, we calculated the prevalence of diseases in each age group and sex using the age distribution of the Korea population. To compare the risk of selected mental disorders among workers in the healthcare industry with those in other industries, we considered age, sex, and income quartile characteristics and conducted propensity scored matching.

**Results:**

In the matching study, workers in healthcare industry had higher odds ratios for mood disorders (1.13, 95% CI: 1.11–1.15), anxiety disorders (1.15, 95% CI: 1.13–1.17), sleep disorders (2.21, 95% CI: 2.18–2.24), and any psychiatric disorders (1.44, 95% CI: 1.43–1.46) than the reference group did. Among workers in healthcare industry, females had higher prevalence of psychiatric disorders than males, but the odds ratios for psychiatric disorders, compared to the reference group, were higher in male workers in healthcare industry than in females.

**Conclusions:**

The prevalence of mood disorders, anxiety disorders, sleep disorders, and all psychiatric disorders for workers in the healthcare industry was higher than that of other Korean workers. The strikingly high prevalence of sleep disorders could be related to the frequent night-shifts in these professions. The high prevalence of mental health problems among workers in healthcare industry is alarming and requires prompt action to protect the health of the “protectors.”

## Background

In modern society, mental illness is an increasingly common problem. The World Health Organization (WHO) estimated the total number of people with depression exceeded 300 million in 2015, and a similar number of people are experiencing anxiety disorders [1]. Depression is the leading cause of global Years Lost due to Disability (YLD), and anxiety disorders were the sixth highest cause in 2015 [2]. These psychiatric disorders are associated with long-term sick leave from the workplace and loss of productivity [3–5].

Mental illness is a major concern in healthcare industries. The mental health of healthcare professionals is especially important, because their mental health is associated with medical errors [6, 7] or decreased performance [8], and these could eventually negatively impact patients’ health. Furthermore, mental health problems experienced by healthcare workers contribute to the high turnover rate [9, 10], which affects the costs of medical institutions through training costs and decreased productivity [11–13]. Combined, these effects put the health of patients at risk.

A number of studies have shown that doctors, nurses, and other healthcare professionals are exposed to psychological distress, such as role conflict, emotional labor, being concerned about medical errors and litigation, as well as experiencing verbal or physical abuse by patients and caregivers or bullying by colleagues [14–17]. In addition, healthcare professionals have a much greater chance of being exposed to long working hours, night work, or shift work. Therefore, they frequently experience sleep problem [18–20]. These work-related stress factors could lead to burnout, and even depression, anxiety disorders, sleep disorders, or other psychiatric disorders [5, 21–27].

However, most of the aforementioned studies have assessed the psychological distress using self-reporting questionnaires. This approach involves limitations, since self-reporting questionnaires do not necessarily accurately reflect the mental health of the participants. Moreover, psychological distress may not always cause a mental disorder due to inter-individual differences in regard to resilience, social support, etc. In fact, a prior study reported that there was a mismatch between high distress levels and clinical depressive disorders or anxiety disorders [28].

Taking these points into account, we aimed to explore the prevalence of mental health disorders in workers in healthcare industry using the actual diagnoses derived from the nationwide Korean National Health Insurance claims data for 2014.

## Methods

### Data source

We used the Korean National Health Insurance (NHI) claims data from January 1 to December 31, 2014 to investigate patients with mental health disorders. The NHI database contains various types of information about healthcare facilities and patients, including the date of visit, total number of patients, diagnoses for claims, prescriptions, admission and discharge, and medical services. Under the health insurance policies, all healthcare facilities in Korea are required to submit the data for medical services they provide to patients to the National Health Insurances Services (NHIS). As the NHIS is prohibited from providing personal identification information to the researchers, information that identifies patients is not included in the data.

### Study population

**Fig. 1:**
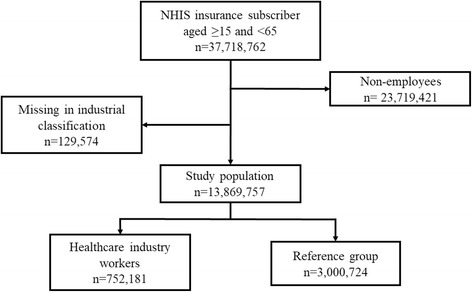
Flow chart of the study population

Participants in this study comprised Korean nationals registered as insured employees in the NHIS between the ages of 15 and 64 as of January 1, 2014. Sex, age, insurance fee (20-class), industry classification, and psychiatric disorders (F00-F99, G47) were extracted from the database subject to this analysis. We classified employees as workers in the healthcare industry based on the NHI data for companies registered with hospitals, clinics, public healthcare, and other medical services. We requested the NHI claims data for patients’ age, sex, primary and secondary diagnosis of disease, date of diagnosis, insurance fee, and industry classification of companies. The insurance fees were calculated by the NHIS according to earned income of employees, or estimated income and property of local subscribers. Industry classification was registered based on the company’s report. After the exclusion of non-employees (*n* = 23,719,421) and participants who were missing an industry classification (*n* = 129,574), a total of 13,869,757 participants were included in our study (Fig. 1).

### Outcome variables

Diagnoses were coded according to the sixth revision of the Korean Standard Classification of Diseases, which is based on tenth revision of the Internal Classification of Diseases. We selected three mental disorders: mood disorders (F30–39), anxiety disorders (F41 and F41.0-F41.9), sleep disorders (F51, F51.0-F51.2, F51.8, F51.9, G47, G47.0, G47.1, G47.2, G47.8, and G47.9), as well as any psychiatric disorders (any of F00-F99 and sleep disorders). We excluded sleepwalking, night terrors, nightmares, sleep apnea, narcolepsy, and cataplexy from sleep disorders, because there is a lack of evidence that those conditions are related to job stress. Any patient who was diagnosed with a primary or secondary mood disorder, anxiety disorder, or sleep disorder during the 1-year period was considered as a case for each disease diagnosed. Patients who had been diagnosed with the same disease multiple times were counted once.

### Prevalence

To estimate the age-standardized prevalence of selected mental health disorders in the total sample of employees and in the workers in healthcare industry, we calculated the prevalence of the disorders according to age group and sex. Korean population structure data from the 2013 resident population and household status, reported by the Ministry of the Interior and Safety of Korea and presented by Statistics Korea [29], were used for the standard population.

### Matching

To compare the risk of selected mental health disorders among workers in the healthcare industry and employees in other industries, we selected the reference group from the entire group of insured employees, excluding those working in the healthcare industry, because there were significant differences in characteristics. To achieve balance, we considered age, sex, and income quartile characteristics and conducted greedy matching based on the propensity score. The propensity score is a balancing score of the distribution of measured covariates and can be used to reduce selection bias in observational studies [30]. After 1:4 matching, a final total of 3,008,724 participants were selected as the reference group.

### Statistical analysis

Prevalence of the selected mental health disorders was calculated for the entire sample of insured employees. Odds ratios for the mental health disorders among the insured healthcare employees were calculated as well. To calculate odds ratios, conditional logistic regression was performed due to the matching strategy used. We used SAS version 9.4 for Windows to perform all statistical analyses, and defined statistical significance as *p*-value < 0.05.

## Results

### Prevalence estimates for selected psychiatric disorders

Among all insured employees, females demonstrated a higher percentage of mood disorders (2%), anxiety disorders (2.26%), sleep disorders (2.1%), and any psychiatric disorders (6.53%) compared to males. The most common disorder was anxiety disorder in both sexes. Among workers in healthcare industry, females had a higher percentage of psychiatric disorders than males, except for sleep disorders. Compared with all insured employees, sleep disorders were the most common problem, and the overall frequency of psychiatric disorders was higher in workers in the healthcare industry (Table 1).

**Table 1:** Prevalence of psychiatric disorders among National Health Insurance service insured employees in Korea (2014)

Prevalence cases	All insured employees						Workers in healthcare industry					
	Both (*n* = 13,869,767)	%	Male (*n* = 8,535,138)	%	Female (n = 5,334,629)	%	Both (*n* = 752,181)	%	Male (*n* = 196,957)	%	Female (*n* = 555,224)	%
Mood disorders^a^	217,752	1.57	111,269	1.30	106,483	2.00	13,709	1.82	3089	1.57	10,620	1.91
Anxiety disorders^b^	250,895	1.81	130,570	1.50	120,325	2.26	15,570	2.07	3528	1.79	12,042	2.17
Sleep disorders^c^	228,119	1.64	116,208	1.36	111,911	2.10	24,965	3.32	6861	3.48	18,104	3.26
Any psychiatric disorders^d^	728,767	5.25	380,223	4.45	348,544	6.53	55,139	7.33	13,512	6.86	41,627	7.50

^a^Mood disorders include diagnosis code of F30~F39 by Korean Standard Classification of Diseases

^b^Anxiety disorders include diagnosis code of F41 and F41.0~F41.9

^c^Sleep disorders include diagnosis code of F51, F51.0~F51.2, F51.8, F51.9, G47, G47.0, G47.1, G47.2, G47.8, and G47.9

^d^Any psychiatric disorders include diagnosis code of any of F00~F99 and sleep disorders

Age-standardized 12-month prevalence estimates of selected disorders among all employees were as follows: mood disorders: 1.69% (95% CI: 1.69–1.70), anxiety disorders: 1.93% (95% CI: 1.92–1.93), sleep disorders: 1.76% (95% CI: 1.75–1.77), and any psychiatric disorders: 5.59% (95% CI: 5.59–5.60).

For workers in healthcare industry, prevalence estimates were higher than those for employees in all industries: mood disorders: 1.93% (95% CI: 1.83–2.03), anxiety disorders: 2.18% (95% CI: 2.08–2.07), sleep disorders: 3.47% (95% CI: 3.39–3.54), and any psychiatric disorders: 7.58% (95% CI: 7.43–7.74). The prevalence of all selected mental disorders among females was higher than that of males in both groups (Table 2).

**Table 2:** Age-standardized prevalence estimate of psychiatric disorders among National Health Insurance service insured employees in Korea (2014)

Age-standardized prevalence estimate (%)	All insured employees						Workers in healthcare industry					
	Both	95% CI	Male	95% CI	Female	95% CI	Both	95% CI	Male	95% CI	Female	95% CI
Mood disorders^a^	1.69	1.68–1.70	1.30	1.29–1.31	2.08	2.07–2.10	1.93	1.83–2.03	1.63	1.45–1.81	2.24	2.17–2.31
Anxiety disorders^b^	1.93	1.92–1.93	1.49	1.48–1.50	2.37	2.36–2.39	2.18	3.39–3.54	1.83	1.66–1.99	2.53	2.46–2.60
Sleep disorders^c^	1.76	1.75–1.77	1.33	1.32–1.34	2.20	2.18–2.21	3.47	2.08–2.27	3.33	3.21–3.45	3.62	3.53–3.71
Any psychiatric disorders^d^	5.59	5.57–5.60	4.38	4.36–4.40	6.83	6.80–6.85	7.58	7.43–7.74	6.77	6.50–7.04	8.43	8.30–8.56

^a^Mood disorders include diagnosis code of F30~F39 by Korean Standard Classification of Diseases

^b^Anxiety disorders include diagnosis code of F41 and F41.0~F41.9

^c^Sleep disorders include diagnosis code of F51, F51.0~F51.2, F51.8, F51.9, G47, G47.0, G47.1, G47.2, G47.8, and G47.9

^d^Any psychiatric disorders include diagnosis code of any of F00~F99 and sleep disorders

### Matched population

After propensity score matching, workers in healthcare industry and those in the reference group were not significantly different with regard to age, income level, and sex (Table 3). The mean age was 37.0 ± 10.5 for healthcare industry workers and 37.1 ± 10.5 for the reference group. The proportion of male workers was 26.18% in both groups. Matching reduced standardized differences for age, sex, and income level.

**Table 3:** Characteristics of employees before and after propensity score matching among study population

Variables	All insured employees		Workers in healthcare industry		Reference group		Standardized difference^a^	
	(*n* = 13,869,767)	%	(n = 752,181)	%	(*n* = 3,008,724)	%	pre-match	post-match
Age							− 0.328	0.000
< 20	100,403	0.72	2578	0.34	10,312	0.34		
20–29	2,424,686	17.48	218,899	29.10	875,596	29.10		
30–39	4,151,253	29.93	239,610	31.86	958,440	31.86		
40–49	3,942,566	28.43	180,096	23.94	720,384	23.94		
50–59	2,721,879	19.62	93,849	12.48	375,396	12.48		
≥60	528,980	3.81	17,149	2.28	68,596	2.28		
Mean	40.4 ± 10.8		37.0 ± 10.5		37.1 ± 10.5			
Income level							−0.056	0.000
Q1	3,471,369	25.03	163,190	21.70	652,760	21.70		
Q2	3,440,340	24.80	247,161	32.86	988,644	32.86		
Q3	3,483,627	25.12	186,525	24.80	746,100	24.80		
Q4	3,474,431	25.05	155,305	20.65	621,220	20.65		
Sex							−0.056	0.000
Male	8,535,138	61.54	196,957	26.18	787,828	26.18		
Female	5,334,629	38.46	555,224	73.82	2,220,896	73.82		

^a^Standardized differences in age was calculated by categorical variables of age group

### Odds ratios for selected psychiatric disorders among workers in healthcare industry

The odds ratios for workers in healthcare industry were higher for mood disorders, anxiety disorders, sleep disorders, and any psychiatric disorders compared to the reference group (Table 4). Risk of sleep disorders was highest in both sexes; odds ratios were 2.21 (95% CI: 2.18–2.24) for the full healthcare sample, and 2.78 (95% CI: 2.69–2.85) for males and 2.05 (95% CI: 2.01–2.09) for females, respectively. For mood disorders, odds ratios were 1.24 (95% CI: 1.19–1.29) for males and 1.10 (95% CI: 1.08–1.12) for females, and for anxiety disorders, 1.19 (95% CI: 1.14–1.23) for males and 1.14 (95% CI: 1.12–1.17) for females, respectively. For any psychiatric disorders, odds ratios were 1.62 (95% CI: 1.59–1.66) for males and 1.39 (95% CI: 1.38–1.41) for females. The odds ratios for mental health disorders were generally higher for male healthcare industry workers than those for female workers, compared with the reference group.

**Table 4:** Odds ratios of selected psychiatric disorders among workers in healthcare industry versus reference group

	Mood disorders^a^			Anxiety disorders^b^			Sleep disorders^c^			Any psychiatric disorders^d^		
	Number of cases (%)	OR^e^	95%CI	Number of cases (%)	OR^e^	95%CI	Number of cases (%)	OR^e^	95%CI	Number of cases (%)	OR^e^	95%CI
Both												
Workers in healthcare industry	13,709 (1.82)	1.13	1.11 - 1.15	15,570 (2.07)	1.15	1.13 - 1.17	24,965 (3.32)	2.21	2.18 - 2.24	55,139 (7.33)	1.44	1.43 - 1.46
Reference group	48,769 (1.62)	1		54,266 (1.80)	1		46,197 (1.54)	1		156,902 (5.21)	1	
Male												
Workers in healthcare industry	3,089 (1.57)	1.24	1.19 - 1.29	3,528 (1.79)	1.19	1.14 - 1.23	6,861 (3.48)	2.78	2.69 - 2.86	13,512 (6.86)	1.62	1.59 - 1.66
Reference group	10,037 (1.27)	1		11,952 (1.52)	1		10,172 (1.29)	1		34,362 (4.36)	1	
Female												
Workers in healthcare industry	10,620 (1.91)	1.10	1.08 - 1.12	12,042 (2.17)	1.14	1.12 - 1.17	18,104 (3.26)	2.05	2.01 - 2.09	41,627 (7.50)	1.39	1.38 - 1.41
Reference group	38,732 (1.74)	1		42,314 (1.91)	1		36,025 (1.62)	1		122,540 (5.52)	1	

^a^Mood disorders includes diagnosis code of F30~F39 by Korean Standard Classification of Diseases

^b^Anxiety disorders includes diagnosis code of F41 and F41.0~F41.9

^c^Sleep disorders includes diagnosis code of F51, F51.0~F51.2, F51.8, F51.9, G47, G47.0, G47.1, G47.2, G47.8, and G47.9

^d^Any psychiatric disorders includes diagnosis code of any of F00~F99 and sleep disorders

^e^No other covariates included in conditional logistic model

## Discussion

This study shows that the prevalence of mood disorders, anxiety disorders, sleep disorders and all psychiatric disorders among workers in healthcare industry was higher than those for workers in other industries. In our study, the reference group was selected from all other industrial workers through propensity score matching method taking age, sex, and income into consideration to investigate the characteristics of workers in the healthcare industry.

In fact, the estimated prevalence of depressive symptoms, anxiety symptoms, and sleep problems in workers in healthcare industry varies among studies and nations. For depressive symptoms, prevalence was estimated as 10% to 28% [8, 31–34], and for anxiety symptoms, prevalence was estimated as 14% to 25% [34–36]. According to the fourth survey of mental disorders, a nationwide sample study of Korean adults (*n* = 5102) conducted in 2016, the 12-month prevalence of all types of mental disorders listed in the fourth edition of the Diagnostic and Statistical Manual of Mental Disorders (DSM-IV) in Korea was 11.9%. Specifically, the prevalence of mental disorders was as follows: in males, 1.3% for mood disorders, 3.8% for anxiety disorders, and 12.2% for any psychiatric disorders; and in females, 2.5% for mood disorders, 7.5% for anxiety disorders, and 11.5% for any psychiatric disorders [37]. With the exception of mood disorders, the prevalence estimates of disorders in our study were generally lower than those from the fourth survey of mental disorders in Korea. Some of these differences may be due to the difference in study participants. The survey included both workers and non-workers who visited hospitals, while our study only included employees. When we investigated the prevalence of psychiatric disorders among all health insurance subscribers, the prevalence was higher than that in the survey population (data not shown). This implies that there is a strong selection bias—particularly healthy worker effect—for relatively good mental health in employed populations.

Some types of hazardous psychological factors have been reported in the healthcare sector. Possible factors explaining the effects of working in the healthcare sector on mental health disorders include time pressure, low levels of support from supervisors and co-workers, heavy workload, sleep deprivation due to night-shift work, uncertainty regarding decisions, and low autonomy. These workplace stressors negatively affect workers’ mental health [15, 23, 32, 38].

According to previous studies, about 30–40% of healthcare workers suffer from burnout [39–41]. Burnout is an outcome of chronic stress, a state of exhaustion combined with doubts about the value of one’s own work and competence [42]. Burnout has previously been reported to be positively correlated with depression [43]. In a job demand-control model, mismatches between workload and job control increase workers’ stress, which could cause a state of anxiety and aggravate exhaustion [44].

Healthcare workers are also often exposed to conflicts or violent situations [34]. Workers who have experienced physical or emotional violence may feel depressed or anxious. In healthcare facilities, workers must keep working in the place where such an event occurred, and this may contribute to workers being reminded of events and re-experiencing anxiety associated with potential threat [45].

Healthcare workers are under pressure to face patients with empathy instead of expressing negative emotion. Thus, healthcare workers should suppress their negative emotion appropriately. This type of work is called emotional labor. Severe emotional labor is also a job stressor related to burnout and mental disorders [46]. A nationwide study reported that suppressing emotion and engaging with complaining customers were related to depression and anxiety [47].

Sleep disorders were more than twice as high in healthcare workers compared to workers in other industries. A number of previous studies concluded that sleep disorders are common for healthcare workers, due to shift and night work, a well-known risk factor of shift work-related sleep disorders [48], which are more frequent in healthcare compared to other industries. With regard to sleep disorders, somnambulism, night terrors, nightmares, sleep apnea, and narcolepsy were excluded based on a specific diagnostic code. There is a possibility, however, that these disorders were still included in the event that they were categorized as a sleep disorder instead of a specific diagnosis.

This study has several limitations. First, as a cross-sectional study, inferences regarding causation are limited and several biases could have affected the results. Healthcare workers suffering from a mental disorder may have quitted their jobs because they need to utilize emotional intelligence to perform their duties. However, we cannot calculate the magnitude of this healthy worker effect. In general, workers in healthcare facilities have higher knowledge of health and physical accessibility to medical services. In psychiatric disorders, despite of higher accessibility, such knowledge may lead them to be more sensitive to the risk of ‘stigmatization’ by their colleagues or supervisors [49, 50]. The bias caused by differences in accessibility could be verified through comparing outcomes with control-diseases such as rheumatic disease and thyroid disease, but we could not confirm it because such diseases are not included in our data.

Second, the NHI claims data were not designed to be used in studies, so there are limitations with the data. For example, the healthcare industry includes several occupations such as medical doctors, nurses, laboratory technicians, and facility maintenance workers. There could be differences in occupational stress factors and estimated prevalence of mental disorders among these workers. However, there is lack of job information in the NHI claims data. According to the Korea Health Industry Statistics System, the percentages of medical personnel employed in hospitals in Korea comprise about 19% medical doctors, 44% nurses, and 37% other workers [51].

Third, medical records not covered by NHI were excluded from the subject of this study. Most people in the general population worry about possible disadvantages in the workplace caused by psychiatric diagnoses, which, in turn, leads to some patients electing not to apply for national health insurance for treatment to avoid a history of such treatment entering into the public record. Consequently, such cases, not included in the NHI data, were not included in this study. Moreover, since the NHI database does not contain family histories of psychiatric disorders, we could not consider the effect of family history.

Lastly, in terms of clinical aspects, we simply defined mental disorder cases as those for the people who had been diagnosed with mental disorder more than one time, and this could not differentiate the severity of disease. Moreover, diagnoses could have been made by physicians with little expertise in psychiatric evaluation. Further research including dosage and duration of medication, hospitalization, and defining cases with more a sophisticated method is needed.

The strength of our study is the large sample size based on one full year of NHI data related to psychiatric disorders with respect to the entire Korean population of insured employees. To the best of our knowledge, there have not yet been studies on a similar topic with such a massive number of participants.

In addition, most previous studies were based on self-reporting questionnaires; thus, it was hard to assure that prevalence of psychiatric disorders was actually higher among workers in the healthcare industry. This study, however, is distinguished from prior studies in that it is based on actual diagnoses by medical doctors, which are claimed subsequently to the NHIS.

## Conclusions

We found the prevalence of mood disorders, anxiety disorders, sleep disorders, and all psychiatric disorders among workers in the healthcare industry was higher compared to workers in other industries in Korea. Therefore, particular attention should be paid to the mental health of workers in healthcare industry. For institutional or governmental mental illness prevention programs, further research is needed on the risk factors and stressors of the actual occupation of healthcare industry workers.
